# Frictional behavior between bone and additively manufactured Ti6Al4V implants is affected by bone density and surface texture under varying loads

**DOI:** 10.1038/s41598-025-25586-0

**Published:** 2025-11-24

**Authors:** Ali Abedi, Farzam Farahmand, Mohammadjavad Salmanimehrjardi, Hasan Nasiri Khonsari

**Affiliations:** Mechanical Engineering Department, Sharif University of Technology, Tehran, Iran

**Keywords:** Cadaver humerus, Quantitative computed tomography, Selective laser melting, Micro surface texture, Macro surface texture, 3-way ANOVA, Friction tailoring, Biomedical engineering, Mechanical engineering, Implants, Fracture repair

## Abstract

Friction at the bone–implant interface is a key factor in the initial stability of cementless orthopedic implants, largely affecting their long-term osseointegration behavior. Yet, there is limited data available for coefficients of friction (COFs) of different combinations of bone qualities and implant surface morphologies, particularly those produced by additive manufacturing techniques. This study measured the interfacial COFs of combinations of three levels of human cancellous bone qualities, characterized based on the BMD, and four Ti6Al4V micro and macro surface textures, produced using selective laser melting (SLM), under three levels of normal stress (0.5, 1.0, and 1.5 MPa), using a reciprocal friction test apparatus. Results indicated a wide range for the COF (0.37–0.95), significantly influenced by the individual effect of each of the metal surface roughness (strong positive: P-value < 0.05 and F-value = 19.12), bone density (moderate negative: P-value < 0.05 and F-value = 7.22) and normal stress (strong negative: P-value < 0.05 and F-value = 40.63), but not the interaction effects. A more variable frictional behavior was observed for both a lower density bone and a sharper-edged metallic texture, presumably due to the higher ploughing inconsistencies involved. The findings highlight the capability of SLM for developing COF-tailored patient-specific implants, based on personalized bone properties and biomechanical considerations.

**Supplementary Information:**

The online version contains supplementary material available at 10.1038/s41598-025-25586-0.

## Introduction

 There is an increasing trend towards cementless orthopedic implants, especially for younger patients, considering their great advantages over the cemented ones, such as reduced risk of aseptic loosening, enhanced surgical efficiency, and lower rate of complications associated with cement wear and failure^1,2^. A critical issue affecting the performance of the cementless orthopedic implants, however, is the strength and stability of the initial fixation, which is purely mechanical^3–5^. To establish a strong and reliable long-term fixation through osseointegration, the micromotion at the bone-implant interface shall be kept minimal, particularly remaining below the commonly suggested threshold of 100 μm^6,7^.

In general, the interfacial micromotion depends upon the surgical technique, the bone quality, the implant’s geometry, interference fit, and surface morphology, as well as the value and direction of the applied loads^8–10^. A lumped friction model can characterize several of these factors using a single term of the coefficient of friction (COF), which describes the mechanical resistance of the bone-implant interface to shear loads. A high COF helps to reduce the bone-implant interfacial micromotion and promote the bone tissue deposition into the implant surface, hence playing a vital role in the osseointegration performance of cementless implants^10^. Nevertheless, there are reports that an excessively high COF might lead to heightened risks of bone infection and wear^11^.

Previous studies on the COF of bone and implant have often examined the frictional behavior of bone with different metal surface morphologies made by traditional surface treatment methods, e.g., polishing, sandblasting, fiber mesh, or bead sintering, plasma spray coating, etc^12–25^. With the advancements of additive manufacturing (AM) technology, it is now possible to build up an implant from metal powder in a layer-by-layer fashion, and produce almost any type of lattice or surface texture^26–28^. This advantage enables tailoring the COF to remain within an optimal narrow range, desired for each specific application. In spite of the rising trend of employing the AM for the production of implants, particularly cementless patient-specific components^29^, there are limited studies concerning the frictional behavior of bone and AM-produced surface morphologies. More specifically, Biemond et al.^30^ and Bartolomeu et al.^31^ have reported the COFs of the Ti6Al4V lattice structures made by electron beam melting (EBM) and selective laser melting (SLM) techniques against human femoral and porcine mandible cortical bones, respectively. However, to the best of our knowledge, no study in the literature has investigated the frictional properties of bone and AM-made surface textures.

Moreover, previous studies have often neglected the effect of the bone quality on its friction with the implant. In most studies^6,15–17,20,21,32^, the species (human, bovine, porcine, synthetic, etc.), type (cortical or cancellous), anatomical site (proximal tibia, distal femur, etc.) and condition (periosteum-intact or -denuded) of the bone specimens are specified, with no quantitative characterization of the bone quality, in terms of porosity or bone mineral density (BMD). An exception is the work of^14^ who measured the porosity of the cancellous bone samples using micro-CT (range: 0.19–0.78) and found a weak positive correlation between the bone porosity and the COF. Also, in the work of^30^ the quality of cortical bone samples, excised from a single cadaveric femur, was characterized in terms of the BMD. However, the range of BMD variation was very small, as expected for cortical bone, resulting in no significant influence on the frictional properties.

The previous research studies have collectively enhanced our understanding of the tribological properties of bone-implant interactions. There is, however, a need for more detailed data on the frictional behavior of different combinations of bone qualities and implant surface morphologies. This is particularly true for Ti6Al4V patient-specific implants made by AM, where the interfacial COF can be tailored via selection of the surface texture in accordance with the personalized bone properties and biomechanical considerations. Moreover, such information helps for a more realistic representation of the bone-implant contact in modeling and simulation studies^33–37^.

Hence, the objective of this study was to measure the interfacial COFs of different combinations of a variety of cancellous bone qualities, characterized based on the BMD, Ti6Al4V surface textures, produced using SLM, and normal load. Moreover, it was aimed to assess the individual and interaction effects of the bone quality, surface texture, and normal load on the COF using statistical measures.

## Materials and methods

### Bone specimens

**Fig. 1:**
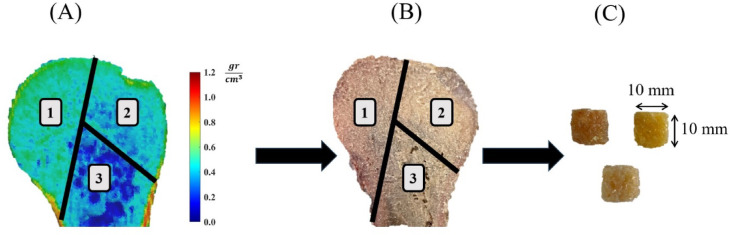
Workflow used to extract cancellous bone specimens with homogenous structures: (**A**) density mapping of QCT data to identify three regions of uniform BMD distribution (1, 2, 3), (**B**) corresponding bone cross-section, illustrating the areas of bone sample extraction, (**C**) excised bone specimens prepared for friction tests.

The experiments of this study were approved by the ethics committee of Tehran University of Medical Science (ethical ID: IR.TUMS.MEDICINE.REC.1400.374). Fourteen humeri bone samples, sourced from human cadavers with an average age of 36.9 years at the time of death (range: 16 to 60 years), were acquired. Informed consent for the use of cadaveric bone specimens was obtained from the legal guardians of the donors, following institutional and ethical guidelines, and all procedures were performed under relevant guidelines and regulations. The bones were cleaned from soft tissues and their metaphyseal regions were scanned using a computed tomography (CT) machine (Brilliance 64, Philips, Germany) with a slice thickness of 0.5 mm and a spatial resolution of 12 Lp/cm. A bone density calibration phantom (QRM- BDC/3 H200, QRM Gmbh, Germany) with three reference materials of zero, 100 and 200 Hounsfield Unit (HU) was placed next to the bone samples during scanning to obtain quantified data. The images were then segmented using an in-house software (Avin Medical Implants, Tehran, Iran) to identify the HU numbers of the voxels, which were then correlated with the BMD using a linear relationship with constants derived from the reference HUs of the calibrating phantom^38^. The distribution of the BMD within each bone sample was then observed, and the homogenous regions of the bone were identified to extract cancellous test specimens with homogenous structures, i.e., uniform BMD distribution (Fig. 1). A total of 48 bone specimens, each measuring 10 mm × 10 mm × 10 mm, were precisely excised from bone samples using a manual precision mitre saw. After sectioning, their dimensions were verified with a profile projector. Only specimens with edge lengths within ± 0.5 mm of the target dimensions were accepted for testing. The bone specimens were categorized into three density groups of equal size: low density (LD), medium density (MD), and high density (HD), with the BMDs in the range of 0.1 to 0.4 gr/cm^3^, 0.4 to 0.7 gr/cm^3^, and 0.7 to 1.0 gr/cm^3^, respectively. Prior to tests, the bone specimens were polished with sandpaper to remove fine particles from their surfaces and kept moist throughout the tests by water spraying.

## Metal specimens

**Fig. 2:**
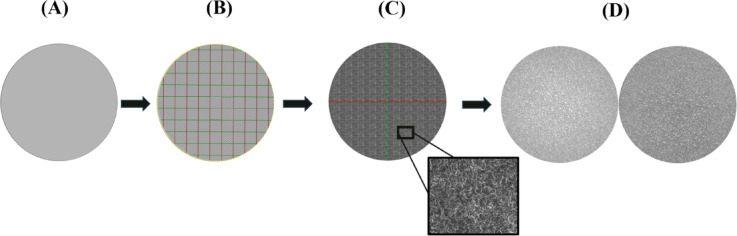
The process of UV (U: horizontal axis, V: vertical axis) mapping for producing consistent surfaces textures by SLM in metal specimens S2 and S3: (**A**) original untextured model; (**B**) application of UV map (**C**) application of SEM image onto the UV map; (**D**) final S2 and S3 textured models exhibiting consistent surface morphologies.

Four disk-shaped metal specimens (diameter: 30 mm; thickness: 8 mm) with different surface conditions were fabricated from Ti6Al4V powder with a particle size range of 15–53 μm (Kyhe Technology, Ningbo, China) using a SLM machine (M100P, Nora Layer Negar Industries, Isfahan, Iran). The first specimen (S1) was fabricated with the typical (as-built) surface conditions of dense titanium parts. The second (S2) and third (S3) specimens were fabricated with two different surface textures to have medium and high roughness. The textures were applied using grayscale surface models, where the dark regions corresponded to valleys and the light regions to peaks, allowing the creation of distinct surface topographies (Fig. 2). The fourth specimen (S4) enjoyed a macro sawtooth surface pattern with 0.8 mm teeth height; this design has been proposed in some recent studies^33,39^ for enhancing frictional contact with high-density cancellous bone.

The SLM procedure was performed with a laser power of 200 W, a hatch spacing of 0.08 mm, a scanning speed of 1200 mm/s, and a layer thickness of 30 μm^40^. After fabrication, the specimens were subjected to stress-relief heat treatment in a vacuum bottom loading furnace (HTBL GR, Caroblite Gero, Derbyshire, UK), by heating up to 600 °C at a rate 10 °C/min, keeping at this temperature for 3 h and then cooling slowly inside the furnace^41^. In order to characterize the surface morphologies of the specimens, their surfaces were imaged using a scanning electron microscope (SEM) (MIRA 3 LMU, Tescan, Brno, Czech Republic) under 8× to 100× magnification. In addition, surface roughness profiles and average surface roughness (Ra) values were obtained using a non-contact laser triangulation profilometer (Fanavari Kahroba, Tehran, Iran). For each specimen, measurements were taken at three randomly selected regions (each approximately 3 × 3 mm) within the central contact area.

## Test procedure

**Fig. 3:**
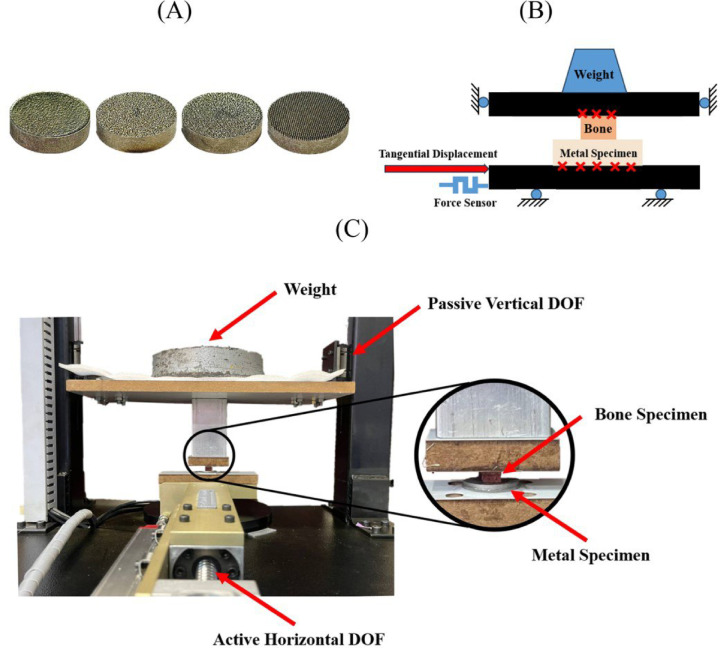
Friction test setup: (**A**) metal specimens (left to right: S1, S2, S3, S4), (**B**) schematics of test apparatus, (**C**) actual test apparatus.

**Fig. 4:**
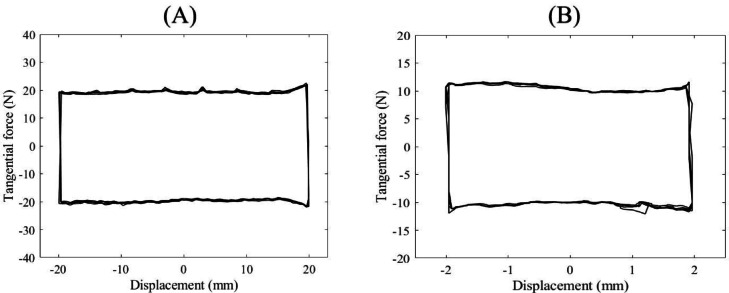
Representative force-displacement diagrams of the friction tests between a UHMPWE sample and a polished stainless-steel surface: (**A**) 200 N normal force, 5 cycles, 20 mm displacement amplitude, (**B**) 100 N normal force, 5 cycles, 20 mm displacement amplitude.

The frictional behavior of various combinations of bone specimens, metal specimens, and applied normal stresses was investigated using a home-made reciprocating friction test apparatus (Fig. 3). The device featured a passive vertical degree of freedom (DOF) to apply normal loads via calibrated weights, and an active horizontal DOF to generate controlled tangential displacements. A load cell (H3-C3, Zemic Europe B.V., Etten-Leur, Netherlands) mounted on the horizontal axis measured the resulting tangential forces. The apparatus was validated through tests involving a UHMWPE polymer sample sliding against a polished stainless-steel plate. Typical load-displacement responses from these validation tests are shown in Fig. 4. Repeated measurements yielded consistent results, with a COF of approximately 0.11, closely aligning with values reported in the literature^24,42,43^.

During the friction tests, first each bone and metal specimen was adhered securely to a wooden holder using a rigid two-part epoxy adhesive (3 M Fast Cure 2-Part Epoxy Metal Filler). To ensure a rigid and secure attachment for the bone specimen, its surface was cautiously dried, before applying the adhesive using a paper towel. The bone and metal specimens were then fixed to the vertical and horizontal DOFs of the apparatus, respectively. For the sawtooth metal specimen, the rotational alignment was carefully adjusted to orient the teeth perpendicular to the direction of motion, whereas accurate alignment was verified using a laser pointer. After each test, the surface of the metal specimen was brushed to remove bone debris and residues, then visually inspected to ensure it had not been damaged. The integrity of the metal surfaces was further verified after all friction tests by re-examining their roughness profiles using the same laser profilometer. For each metal specimen, surface scans were performed at three randomly selected regions (each approximately 3 × 3 mm) within the central contact area. The post-test roughness measurements (Ra) were compared with the corresponding pre-test values which revealed no measurable changes in surface topography (Appendix A). Furthermore, no signs of surface scratching or adherent bone debris were observed in the post-test profiles.

Before the main friction tests, some pilot tests were performed to identify and address the potential problems and refine the test procedure. In pilot tests, pairs of the metal specimens and a number of extra bone specimens were subjected to different normal loads and a displacement amplitude of 20 mm for three cycles. Each main friction test was performed in three steps for each pair of bone and metal specimens. In the first step, while a normal load of 50 N (0.5 MPa normal stress) was applied to the vertical axis, one complete cycle of reciprocal displacement (amplitude: 4 mm; rate: 0.1 mm/s) was implemented by the horizontal DOF, and the resulting tangential force was continuously recorded. In the second and third steps, the normal load was increased to 100 N (1.0 MPa normal stress), and 150 N (1.5 MPa normal stress), respectively, and the same procedure was repeated. The tangential force-horizontal displacement diagram, obtained at each step of each test, was then analyzed, and the COF was calculated as the average tangential force divided by the normal force. In this way, the calculated COF represented the kinetic frictional behavior associated with cyclic reciprocating micromotion at the bone–implant interface under repetitive physiological loading conditions, such as those experienced during gait^15,44^.

## Statistics

For each test group, consisting of four bone samples of the same quality (LD/MD/HD) engaged with a specific metal surface (S1/S2/S3/S4) under a specific normal stress (0.5/1.0/1.5 MPa), the COFs were averaged and described as means (standard deviation). The Kolmogorov-Smirnov (K-S) test was used to assess the normality of data distributions across different test groups, which confirmed that the data were normally distributed in all groups. The three-way ANOVA was utilized for comparative statistical analysis to examine the influence of the bone quality, metal surface, and normal stress on the COF. In case of significant results for ANOVA, post hoc analysis, based on the Tukey test, was performed to determine whether there is a difference between the mean of every possible pair of all groups. The level of significance was considered as 0.05 and the ANOVA and post hoc analysis were performed using SPSS software (Version 26.0, IBM Corp., Armonk, NY, USA; https://www.ibm.com/products/spss-statistics).

## Results

**Fig. 5:**
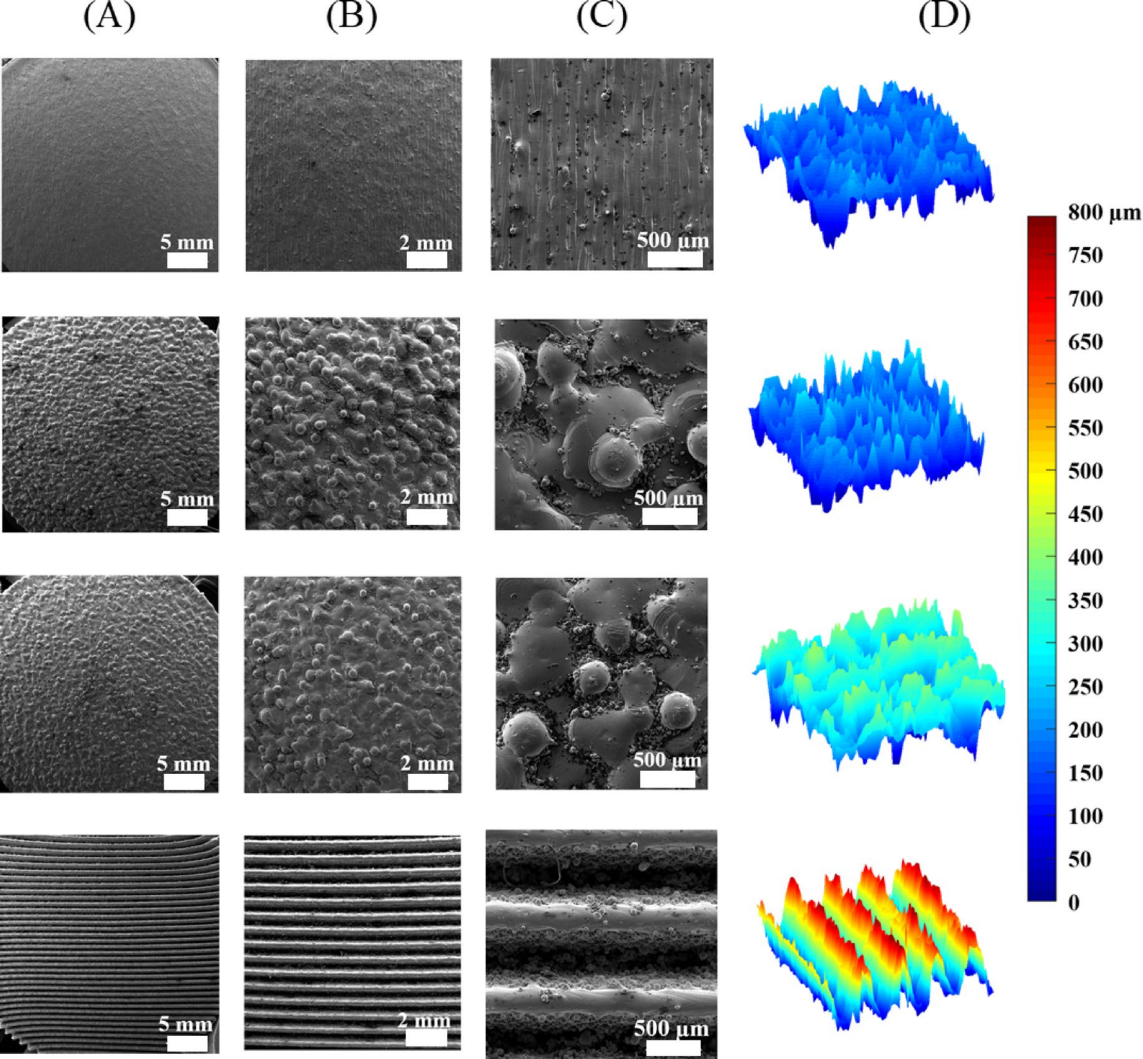
Characterization of the surface morphologies of the metal specimens: (**A**-**C**) SEM images of different magnifications, (**D**) surface roughness profiles. First row: S1 solid non-textured specimen, Second row: S2 medium roughness textured specimen, Third row: S3 high roughness textured specimen, Fourth row: S4 sawtooth surface specimen.

The SEM images and representative roughness profiles of the surfaces of the SLM-fabricated heat-treated specimens are illustrated in Fig. 5. The solid non-textured specimen, S1, exhibited minimal asperities and a relatively low surface roughness (Ra = 7.8 ± 1.1 μm). The surface textured specimens, S2 and S3, revealed uniform distributions of large asperities, causing a substantial increase in the average surface roughness values (Ra = 34.9 ± 4.9 μm for S2 and Ra = 64.9 ± 13.5 μm for S3). Moreover, their surfaces were bonded with large quantities of partially melted Ti particles. The surface of the S4 specimen enjoyed a uniform, well-arranged macro feature morphology of 0.8 mm sawtooth, with little sign of partially melted particles.

**Fig. 6:**
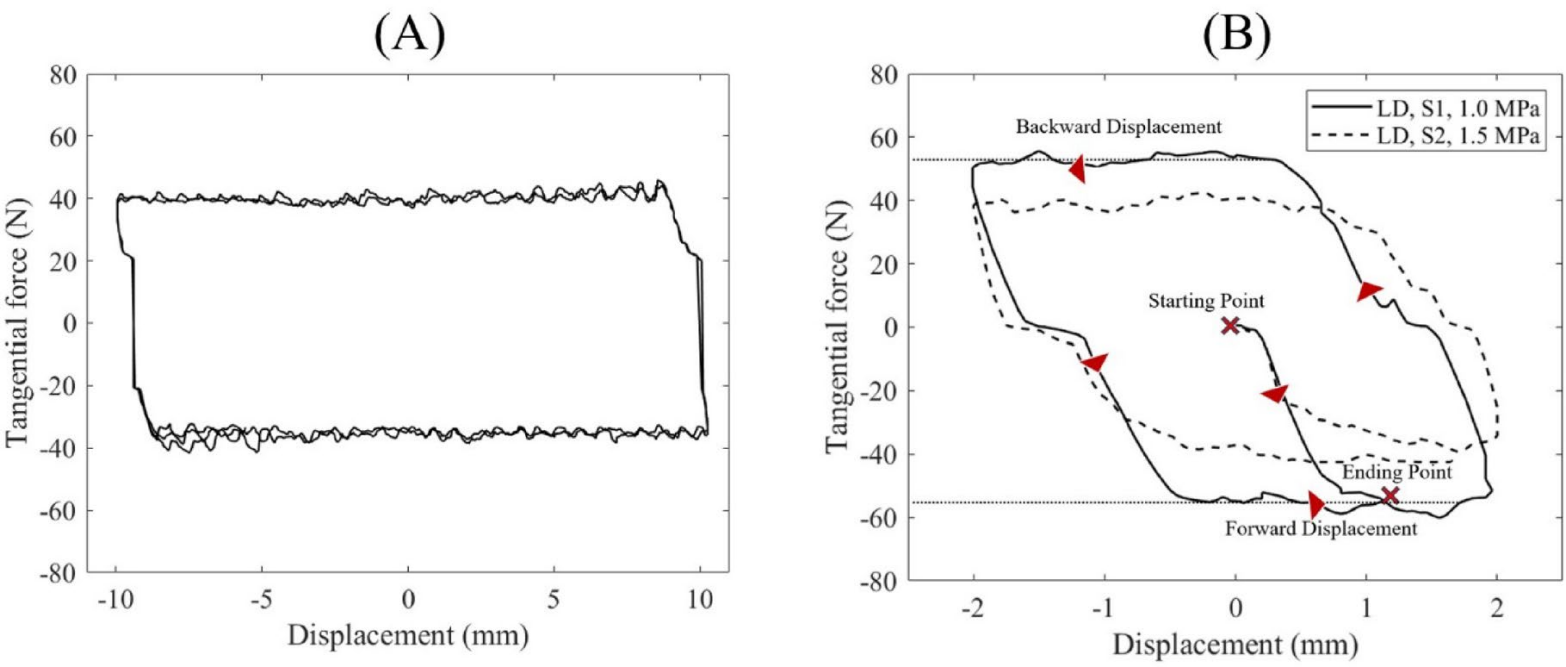
Typical force-displacement diagrams of the friction tests (**A**) representative pilot friction test (three cycles and 10 mm amplitude) between LD bone specimen and S2 metal specimen under 1.5 MPa normal stress. (**B**) main friction tests (one cycle and 2 mm amplitude) between LD bone specimen and S1 metal specimen under 1.0 MPa normal stress, and between LD bone and S2 metal specimen under 1.5 MPa stress.

Sample force–displacement diagrams from both the pilot and main friction tests are shown in Fig. 6. In all tests, the tangential force exhibited minimal fluctuations and remained nearly constant during both forward and backward displacement phases. The pilot test results (Fig. 6A) demonstrated consistent force–displacement loops across three cycles, with a stable tangential force throughout the 20 mm displacement course. Based on this observation, the main tests were limited to a single cycle with a reduced displacement amplitude of 4 mm to minimize potential damage to the bone specimens. As illustrated in Fig. 6B, the mean tangential forces recorded during friction testing between the LD bone specimen and the S1 metal specimen under 1.0 MPa normal stress, and between the LD bone and the S2 metal specimen under 1.5 MPa stress, were 53.50 N and 40.43 N, respectively, which correspond to COFs of 0.54 and 0.27.

The mean COFs of different combinations of the metal specimens (S1/S2/S3/S4), bone samples (LD/MD/HD), and normal stresses (0.5/1.0/1.5 MPa) are indicated in Table 1, along with the previous reports for the COFs between human cancellous bone and Ti6Al4V surfaces. The ranges of variations of the mean COFs were 0.37–0.65 for S1, 0.43–0.90 for S2, 0.50–0.95 for S3, and 0.37–0.93 for S4, where the maximum values were associated with lower bone densities and smaller normal stresses, in general.

**Table 1:** Means (standard deviations) of COFs of different combinations of metal specimens, bone specimens and normal stresses found in present study (bold), along with previous reports for the COFs between cancellous bone and Ti6Al4V surfaces.

Ti6Al4V Surface Type	Ra (µm) (Process)	Bone type (V_f_, A_f_ %) (D $\:\frac{\varvec{g}\varvec{r}}{{\varvec{c}\varvec{m}}^{3}}$)	Normal Stress (MPa)	Mean COF (SD)	Ref
Polished	0.11	Femur	0.1–12	0.16 (0.05)	^17^
	N/A	Femur (V_f_= 8.7–21)	0.15–1	0.19 (0.04)	^14^
	0.1 ± 0.0	Femur (A_f_ = 25–56)	0.1–2.5	0.42	^15^
Micro-Featured	**7.8 ± 1.1** **(SLM solid: S1)**	**Humorous** **(D = 0.1–0.4)**	**0.5/1.0/1.5**	**0.53 (0.12)/0.54 (0.12)/0.37 (0.15)**	**Current study**
		**Humorous** **(D = 0.4–0.7)**		**0.65 (0.10)/0.58 (0.05)/0.55 (0.06)**	**Current study**
		**Humorous** **(D = 0.7–1.0)**		**0.53 (0.05)/0.48 (0.05)/0.43 (0.05)**	**Current study**
	**34.9 ± 4.9** **(SLM-textured: S2)**	**Humorous** **(D = 0.1–0.4)**	**0.5/1.0/1.5**	**0.90 (0.10)/0.67 (0.12)/0.43 (0.25)**	**Current study**
		**Humorous** **(D = 0.4–0.7)**		**0.80 (0.06)/0.62 (0.12)/0.52 (0.18)**	**Current study**
		**Humorous** **(D = 0.7–1.0)**		**0.70 (0.08)/0.55 (0.17)/0.50 (0.14)**	**Current study**
	**64.9 ± 13.5** **(SLM-textured: S3)**	**Humorous** **(D = 0.1–0.4)**	**0.5/1.0/1.5**	**0.90 (0.10)/0.73 (0.21)/0.50 (0.26)**	**Current study**
		**Humorous** **(D = 0.4–0.7)**		**0.95 (0.08)/0.82 (0.08)/0.72 (0.04)**	**Current study**
		**Humorous** **(D = 0.7–1.0)**		**0.88 (0.05)/0.75 (0.10)/0.58 (0.13)**	**Current study**
Macro-Featured	(Lattice)	Femur (V_f_= 8.7–21)	0.15 − 1	0.90 (0.04)	^14^
	**(SLM-Sawtooth: S4)**	**Humorous** **(D = 0.1–0.4)**	**0.5/1.0/1.5**	**0.93 (0.06)/0.77 (0.12)/0.67 (0.15)**	**Current study**
		**Humorous** **(D = 0.4–0.7)**		**0.87 (0.24)/0.73 (0.18)/0.63 (0.23)**	**Current study**
		**Humorous** **(D = 0.7–1.0)**		**0.83 (0.12)/0.57 (0.15)/0.37 (0.15)**	**Current study**
Thermo-chemical Coated	41.2 ± 1.4 (HA)	Femur (A_f_ = 25–56%)	0.1–2.5	0.82	^15^
	53.0 ± 6.2 (HA)		0.1–2.5	0.86	^15^
	N/A (Titanium)	Femur (V_f_= 8.7–21%)	0.15–1	0.74 (0.04)	^14^
Thermo-mechanical Coated	N/A (Fiber meshed)	Tibia	0.1–0.4	0.52 (0.07) − 0.44 (0.07)	^24^
			0.1/0.15/0.25	0.64 (0.09)/0.62 (0.09)/0.63 (0.07)	^25^
	N/A (Beaded)	Tibia	0.1/0.25	0.68 (0.1)/0.68 (0.09)	^23^
	32.6 (Beaded)	Femur	0.1–6	0.86 (0.02)	^17^
	133 (Flaked)	Femur	0.1–6	1.08 (0.04)	^17^

**V**_**f**_: Volume fraction (bone tissue volume/total volume); A_f_: Area fraction (bone tissue area/total area); D: Bone mineral density; HA: Hydroxyapatite.

**Fig. 7:**
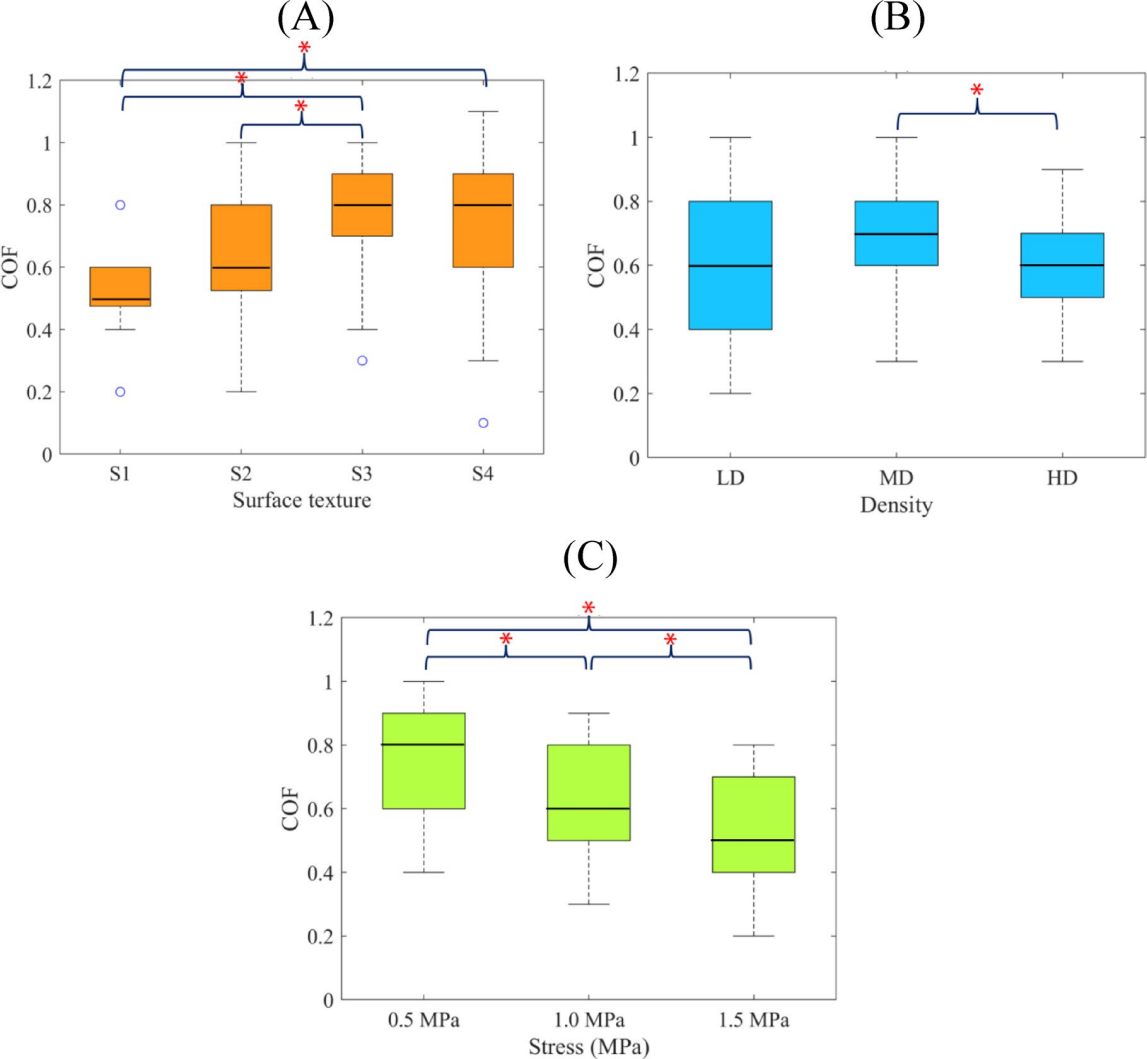
Results of post hoc analysis for individual effects of the variables on COF: (**A**) metal surface texture, (**B**) bone mineral density, (**C**) normal stress. Asterisk indicates a statistically significant difference between groups (*p* < 0.05).

The results of the three-way ANOVA (Table 2) indicated that all variables, i.e., bone quality, metal surface, and normal stress, had significant individual effects on the COF, but their combined effects (interaction effects) were not significant. The F-value results (Table 2) implied that among the three variables, the normal stress, followed by the metal surface, was most impactful. The post hoc analysis indicated that the COF increased consistently with the increase of the metal surface roughness (Fig. 7A) and decrease of the normal stress (Fig. 7C) (*p* < 0.05). The COF of the sawtooth surface with the bone specimens, however, exhibited a large variability and was significantly higher than that of the low roughness metal surface, S1, only (*p* < 0.05). Also, the effect of the bone quality on the COF was not consistent; while the COF of the MD bone was significantly higher than that of the HD bone (*p* < 0.05), it was highly variable for the LD bone and not significantly different from those of the MD and HD (Fig. 7B).

**Table 2:** Results of the three-way ANOVA for individual and interaction effects of variables on COF.

Source	F-Value	*P*-Value
Bone Density	7.22	0.001
Metal Surface	19.12	0.000
Normal Stress	40.63	0.000
Density*Surface	1.70	0.129
Density* Stress	0.85	0.494
Surface * Stress	1.39	0.225
Density*Surface * Stress	0.47	0.930

## Discussion

This study provided a comprehensive assessment of the frictional behavior at the bone–implant interface, involving various combinations of human cancellous bone qualities, quantitatively categorized by BMD, and Ti6Al4V surface morphologies, fabricated via SLM. Unlike previous research, which often neglected the bone quality or used traditionally manufactured implants, this work investigated the independent and combined effects of bone density, SLM fabricated surface textures (micro- and macro-scale), and physiological loading levels (up to 1.5 MPa) on the COF. Notably, the bone specimens were harvested from the proximal humerus, an anatomical site rarely studied in implant fixation research, which offers a broad range of bone density and enables the extraction of structurally homogeneous specimens across LD, MD, and HD groups.

The mean COF results (Table 1) revealed a wide range, from 0.37 to 0.95, depending on the combination of the surface morphology, bone quality, and normal stress. The minimum COF was associated with the pair of the LD bone/S1 metal specimen under 1.5 MPa stress, and the maximum with the pair of the MD bone/S3 metal specimen under 0.5 MPa stress. Our results are in reasonable agreement with the data reported in the literature (Table 1) for similar conditions. For instance, the COF results for the S1 specimen (range of means: 0.37 to 0.65) are slightly larger than the results reported by de Vries et al.^15^ for the Ti6Al4V polished surface (mean: 0.42), which is not unexpected. Also, the COFs of S2 and S3 specimens are in the same range reported previously for thermo-mechanical coated (fiber mesh and bead sintered) Ti6Al4V surfaces^14–16,22–25^. Finally, the COF results for the S4 sawtooth specimen under 0.5 MPa stress (range: 0.88–0.95) are close to the reports of Biemond et al.^30^ for the Ti6Al4V lattice structures under similar loading (mean: 0.90). Our results, however, provide more detail information on the individual and combined effects of the bone density, surface morphology and normal load on the COF. This information can help to improve the design of patient-specific cementless implants by tailoring the COF based on personalized bone properties. It also enables a more realistic representation of the bone-implant contact in modeling and simulation studies.

The ANOVA and post hoc results (Table 2; Fig. 7A) indicated that the metal surface roughness had a strong positive influence on the COF, which is in agreement with the reports of previous studies^16–19^. In particular, the COF increased gradually and consistently by applying textures with higher degrees of harshness to the metal surface (Fig. 7A). As illustrated in the SEM images and roughness profiles of the metal surfaces (Fig. 4), all SLM-made specimens had well-defined and consistent surface topographies, including uniformly distributed small, medium and large asperities in S1, S2 and S3 specimens, respectively, and a sawtooth surface pattern in S4 specimen. This observation suggests that the SLM method is capable of producing a wide variety of micro- and macro-morphological features on the surface of Ti6Al4V implants, in order to deliver any desired frictional behavior effectively.

Nevertheless, the surface textured specimens, S2 and S3, involved large quantities of partially melted Ti particles bonded to their surfaces (Fig. 4). This incident is not unexpected considering the laser-based layer-by-layer melting approach of particles in the SLM, which can cause a stair-stepping effect^45^. In implant applications, such bonded particles are typically removed through additional post-processing treatments, such as sandblasting, since their release into the biological environment may trigger adverse effects such as inflammation and bioactivity deterioration^40,46^. However, recent reports suggest that these partially melted particles may promote osteogenic differentiation of human osteoblasts on as-printed surfaces^47,48^, indicating that the direct use of as-built surfaces, without post-processing that alters surface morphology, could be a promising approach for certain implant applications.

The sawtooth surface specimen, S4, exhibited a highly variable frictional behavior and produced a significantly larger COF, only in comparison with the non-textured specimen, S1 (Fig. 7A). This finding is somehow against the previous assumption that sharp cutting-edge textures can penetrate into the bone and enhance the ploughing frictional effects compared to the textured surface morphologies, and suggests that the small lateral contact area between the teeth and bone might counterbalance the effect of a deeper indentation^15^. This justification is approved by the higher variation of the COF of all textured specimens, in comparison with the non-textured S1 specimen (Fig. 7A), which is attributed to a more inconsistent mechanical interaction between large surface asperities and bone.

The results also indicated a moderate negative influence of the bone density on the COF, regardless of the metallic surface characteristic and stress level (Table 2; Fig. 7B). The COF was significantly smaller in HD bone specimens in comparison with the MD (*p* < 0.05), but not different between the other bone groups. This observation is in agreement with the results reported by Dannaway et al.^14^ and might be linked to the lower degree of penetration of metal surface asperities into a solid, strong bone surface. In fact, the sawtooth texture was designed originally to enhance the mechanical interaction between the surface of a metallic suture anchor and such bone^33,39^. However, as indicated in Table 2, the interaction effect of the bone density and the surface morphology was not significant, suggesting the inefficacy of this design. For the LD bone, the COF was highly variable (Fig. 7B); this behavior might be attributed again to the inconsistencies involved in the mechanical interaction of the metal surface asperities and a highly porous bone surface. Nevertheless, the significant effect of the bone quality on the bone-implant frictional behavior underscores the importance of tailoring the implant surface morphology in accordance with the personalized bone properties.

Another interesting finding of this study was the significant effect of the loading on the bone-implant frictional behavior, which has been neglected in most previous studies^15–17,19,30,32,49,50^. Moreover, the stress level has often been kept small, up to 0.5 MPa^15–17,30,32,49^, which is much lower than the contact pressures found at the interface of the bone and press-fit implants^51^. The results of our study for 0.5, 1.0, and 1.5 MPa normal stress indicated a strong negative influence of the normal stress on the COF (Table 2; Fig. 7C), similar to the findings of^44^. This behavior is thought to be caused by the reduced ploughing effects due to the plastic deformation of the cancellous bone under large normal stresses^15^. Nevertheless, the formation of detached bone particles during intense plowing, which roll between the metal and bone, might also be responsible for the decrease of the COF. Further research, using advanced imaging techniques, can help to get more insight into the mechanisms involved in this effect.

Our study suffers from some limitations, which shall be addressed in future investigations. First of all, the number of bone specimens was limited to 48, which resulted in four samples of the same quality (LD/MD/HD) in each test group. This limitation might have contributed to the insignificant combined effects found for the bone density, metal surface characteristics, and normal stress (Table 2). In particular, considering the p-value observed for the interaction of the bone density and surface texture, a significant effect might appear if a larger number of bone specimens are tested.

Furthermore, the maximum normal stress applied in our tests was limited to 1.5 MPa. While this stress level is adequate for representing bone–implant interactions experienced by cancellous tissues, such as the humeral metaphysis, the interfaces involving cortical bone, particularly in the lower extremities, may be subjected to considerably higher loads. For instance, contact pressures at the interface between the femoral stem and bone shaft in cementless total hip arthroplasty have been reported to reach up to 25 MPa for a 0.1 mm interference fit^51^. To investigate frictional behavior under such elevated loading conditions, future tests should employ strong cortical bone specimens from the lower extremities to prevent crushing, while also limiting the displacement amplitude to minimize interference from bone particle detachment.

Finally, this study focused exclusively on pre-wear conditions and did not incorporate post-test surface or wear analyses beyond roughness profiling. Although post-test measurements confirmed that the metal surfaces remained undamaged, with no measurable changes in surface morphology or signs of adherent bone debris (Appendix A), these evaluations were limited to laser profilometry. More detailed surface characterizations—such as high-resolution scanning electron microscopy (SEM), compositional analysis, or higher-resolution profilometry—could provide deeper insights into wear behavior and the tribological mechanisms at the bone–implant interface. Notably, in our study the same set of metal specimens was reused across multiple friction tests. While the short displacement amplitudes and limited number of cycles were expected to prevent surface degradation, this reuse, coupled with limited post-test evaluation, restricts the ability to fully exclude micro-scale wear or residual bone contamination. Future studies are therefore encouraged to use fresh metal specimens for each test condition and incorporate more comprehensive post-test surface analyses to ensure accurate interpretation of frictional behavior at the bone–implant interface.

## Conclusion

The frictional behavior of the bone-implant interface is affected by bone quality, metal surface characteristics, and loading. The COF ranged between 0.37 and 0.95 for combinations of a variety of Ti6Al4V surface morphologies, cancellous bone qualities, and normal loads. It was found that the COF is influenced significantly by the individual effect of each of the metal surface roughness (strong positive), bone density (moderate negative), and normal stress (strong negative), but not the interaction effects. A more variable frictional behavior was observed for both a lower density bone and a sharper-edged metallic texture, presumably due to the inconsistencies involved in ploughing of a highly porous bone surface and/or large metal asperities. The SLM method was found to be capable of producing a wide variety of micro- and macro-morphological features, with well-defined and consistent topographies. This capability is promising for developing advanced COF-tailored patient-specific implants by applying surface morphologies in accordance with the personalized bone properties and biomechanical considerations.

## Supplementary Information

Below is the link to the electronic supplementary material.

Supplementary Material 1

## Data Availability

The supplementary files provide sample datasets for four different surface conditions. As the raw experimental data were collected and processed within a laboratory system, direct access to the entire dataset is not straightforward. However, all datasets are available from AA upon reasonable request via email.

## Appendix A. Post-Test surface roughness assessment

To confirm that the friction tests did not alter the surface characteristics of the metal specimens, surface roughness measurements (Ra) were re-evaluated using the same non-contact laser profilometer (Fanavari Kahroba, Tehran, Iran) employed in pre-test characterization. For each specimen, three randomly selected regions (approximately 3 × 3 mm) were scanned within the central contact area after all tests were completed. The mean post-test roughness values were then compared with the pre-test values.

**Table:**

Specimen ID	Surface type	Pre-test Ra (µm)	Post-test Ra (µm)
S1	Solid non-textured (as-built)	7.8 ± 1.1	7.7 ± 1.0
S2	Textured (medium roughness)	34.9 ± 4.9	35.1 ± 5.0
S3	Textured (high roughness)	64.9 ± 13.5	65.2 ± 13.1
S4	Sawtooth surface	-	-

**Note**: Post-test measurements for the S4 macro-textured specimen were not performed due to its geometric complexity and large-scale features, which fall outside the resolution capability of the profilometer.

Across all measurable surfaces, the post-test profiles showed no significant deviation from pre-test values. Moreover, visual inspection of the surface scans revealed no evidence of surface scratching, plastic deformation, or adherent bone debris.
